# The Demethoxy Derivatives of Curcumin Exhibit Greater Differentiation Suppression in 3T3-L1 Adipocytes Than Curcumin: A Mechanistic Study of Adipogenesis and Molecular Docking

**DOI:** 10.3390/biom11071025

**Published:** 2021-07-14

**Authors:** Ahmed Alalaiwe, Jia-You Fang, Hsien-Ju Lee, Chun-Hui Chiu, Ching-Yun Hsu

**Affiliations:** 1 Department of Pharmaceutics, College of Pharmacy, Prince Sattam Bin Abdulaziz University, Al Kharj 11942, Saudi Arabia; alalaiwe@gmail.com; 2 Pharmaceutics Laboratory, Graduate Institute of Natural Products, Chang Gung University, Kweishan, Taoyuan 333, Taiwan; fajy@mail.cgu.edu.tw (J.-Y.F.); daxialeetw@gmail.com (H.-J.L.); 3 Research Center for Food and Cosmetic Safety and Research Center for Chinese Herbal Medicine, Chang Gung University of Science and Technology, Kweishan, Taoyuan 333, Taiwan; chchiu@mail.cgust.edu.tw; 4 Department of Anesthesiology, Chang Gung Memorial Hospital, Kweishan, Taoyuan 333, Taiwan; 5 Graduate Institute of Health Industry Technology, Chang Gung University of Science and Technology, Kweishan, Taoyuan 333, Taiwan; 6 Department of Nutrition and Health Sciences, Chang Gung University of Science and Technology, Kweishan, Taoyuan 333, Taiwan

**Keywords:** curcuminoid, demethoxycurcumin, bisdemethoxycurcumin, adipocyte, anti-adipogenesis, transcription factor

## Abstract

Curcumin is a known anti-adipogenic agent for alleviating obesity and related disorders. Comprehensive comparisons of the anti-adipogenic activity of curcumin with other curcuminoids is minimal. This study compared adipogenesis inhibition with curcumin, demethoxycurcumin (DMC), and bisdemethoxycurcumin (BDMC), and their underlying mechanisms. We differentiated 3T3-L1 cells in the presence of curcuminoids, to determine lipid accumulation and triglyceride (TG) production. The expression of adipogenic transcription factors and lipogenic proteins was analyzed by Western blot. A significant reduction in Oil red O (ORO) staining was observed in the cells treated with curcuminoids at 20 μM. Inhibition was increased in the order of curcumin < DMC < BDMC. A similar trend was observed in the detection of intracellular TG. Curcuminoids suppressed differentiation by downregulating the expression of peroxisome proliferator-activated receptor γ (PPARγ) and CCAAT/enhancer-binding protein α (C/EBPα), leading to the downregulation of the lipogenic enzymes acetyl-CoA carboxylase (ACC) and fatty acid synthase (FAS). AMP-activated protein kinase α (AMPKα) phosphorylation was also activated by BDMC. Curcuminoids reduced the release of proinflammatory cytokines and leptin in 3T3-L1 cells in a dose-dependent manner, with BDMC showing the greatest potency. BDMC at 20 μM significantly decreased leptin by 72% compared with differentiated controls. Molecular docking computation indicated that curcuminoids, despite having structural similarity, had different interaction positions to PPARγ, C/EBPα, and ACC. The docking profiles suggested a possible interaction of curcuminoids with C/EBPα and ACC, to directly inhibit their expression.

## 1. Introduction

Obesity influences one-third of adults globally, and represents a primary health problem [1]. The increasing prevalence of overweight children is also a concern. Obesity can shorten the life span and disrupt the function of many organs [2]. Collectively, this increases the risk of chronic obesity-related co-morbidities, including diabetes, hypertension, dyslipidemia, dyspnoea, fatty liver, cancer, and poor mental health [3]. The hyperplasia and hypertrophy of adipocytes are important characteristics of obesity. Adipocytes are the primary constituent of adipose tissue and store triacylglycerol, which plays a crucial role in maintaining energy balance. In addition to storing fat, adipocytes are considered to influence endocrine cells, which secrete numerous bioactive peptides called adipokines, and has a profound impact on the metabolic homeostasis of other tissues [4]. The inhibition of adipogenesis, by reducing preadipocyte differentiation, is a promising therapeutic target to prevent or improve obesity and its associated comorbid conditions. Several anti-obesity drugs have been approved in the past ten years; however, most of them have been withdrawn due to adverse effects. For instance, amphetamine, rimonabant, and sibutramine have been shown to increase the risk of myocardial infarction and psychiatric disorder [5]. Their long-term success has also been questioned, considering weight regain after treatment discontinuation.

**Figure 1:**
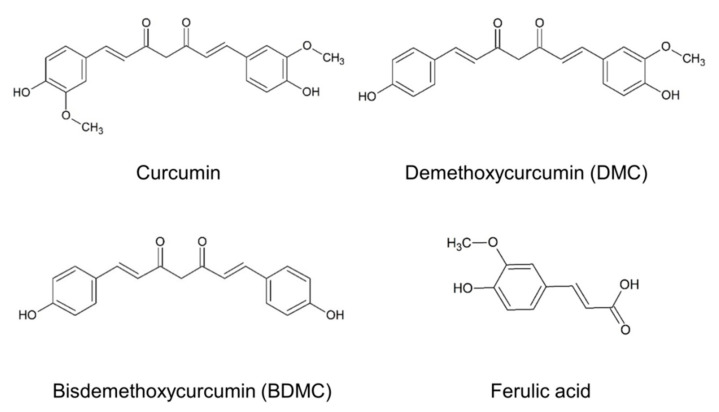
The chemical structures of curcumin, demethoxycurcumin (DMC), bisdemethoxycurcumin (BDMC), and ferulic acid.

Natural products and phytochemicals are potential agents that ameliorate obesity via adipogenesis suppression, lipolysis enhancement, adipocyte hypertrophy inhibition, and fat cell apoptosis [6]. Herbal extracts, or their isolated compounds, can provide nutritional intervention for obesity management, due to their low cost and favorable side-effect profile. Among these phytochemicals, turmeric (*Curcuma longa*) has demonstrated beneficial activity in obesity and diabetes [7]. Curcumin is a linear diarylheptanoid that is primarily found in turmeric (Figure 1). Curcumin has been shown to have anti-inflammatory, antioxidant, neuroprotective, and chemopreventive properties [8], possibly by inhibiting preadipocyte differentiation and inflammation, as well as activating cellular antioxidants [9]. Clinical evidence supports the bioactivity of curcumin in promoting weight loss and reducing the triglyceride (TG) levels in patients with diabetes [10,11]. The pharmacological activity of turmeric is primarily derived from curcuminoids, which are a mixture of curcumin and its demethoxy derivatives, including demethoxycurcumin (DMC) and bisdemethoxycurcumin (BDMC). These polyphenols share the same structure of two benzenemethoxyl rings connected by an unsaturated chain (Figure 1). DMC and BDMC lack one and two methoxy moieties in the aromatic rings of curcumin, respectively. Antioxidant, anticancer, and neuroprotective potential of the demethoxy analogs of curcumin have been reported [11]. Although curcumin has been shown to improve obesity and the related syndromes, there is a paucity of data regarding the anti-obesity activity of DMC and BDMC. Lai et al. [12] demonstrated that BDMC inhibited the development of obesity in high-fat diet-fed mice. We aimed to systematically compare the inhibitory activity of curcumin and its demethoxy analogs against adipogenesis. The underlying mechanisms of the adipogenesis suppression by these compounds were also explored, using a cell-based study and computational molecular docking. Adipogenesis is a process that is involved in the differentiation of preadipocytes into mature adipocytes via intracellular lipid deposition. We employed an in vitro model with 3T3-L1 preadipocytes, to elucidate the molecular mechanisms of adipogenesis regulation by curcuminoids. Differentiation of the 3T3-L1 preadipocyte fibroblast cell line into mature adipocytes is one of the most commonly used in vitro models to study adipose tissue biology. Adipogenesis is controlled by the family of peroxisome proliferator-activated receptors (PPARs) and CCAAT/enhancer-binding proteins (C/EBPs). In addition, lipogenic enzymes such as acetyl-CoA carboxylase (ACC) and fatty acid synthase (FAS) are essential for anabolic lipogenesis [13]. These pathways offer the potential of therapeutic targets for combating obesity. An understanding of the underlying mechanisms regulating adipogenesis is helpful for examining obesity prevention and therapy with curcuminoids.

## 2. Materials and Methods

### 2.1. Analysis of Curcuminoids in Turmeric Extract

The extract from turmeric rhizome (BCM-95) was supplied by Arjuna Natural (Kerala, India) and dissolved in ethanol. High-performance liquid chromatography (HPLC) was used to determine the amounts of curcumin, DMC, and BDMC in the extract. The HPLC system was from Hitachi Primaide (Tokyo, Japan) with an Agilent Zorbax SB-C18 column (Santa Clara, CA, USA). The mobile phase consisted of a mixture of acetonitrile and water containing 2% acetic acid (6:4). The flow rate and detection wavelength were set at 2 mL/min and 420 nm, respectively.

### 2.2. Molecular Modeling and Docking

The structures of curcuminoids were sketched by Discovery Studio version 4.1 workstation (Accelrys, San Diego, CA, USA). The physicochemical characteristics of these molecules estimated by Discovery Studio included molecular volume, predicted oil/water partition coefficient (Alog *P*), total polarity surface, hydrogen bond acceptor number, and hydrogen bond donor number. The crystal structures of PPARγ (protein data bank PDB ID: 1ZGY), C/EBPα (PDB ID: 1NWQ), and ACC (PDB ID: 2DN8) were downloaded from the RCSB Protein Data Bank (www.rcsb.org). The molecular docking between these proteins and curcuminoids was performed by Discovery Studio. The negative CDOCKER energy was computed after conducting the docking simulation of the curcuminoids with the proteins to assess possible interactions.

### 2.3. Cell Culture and Differentiation

The 3T3-L1 preadipocytes were purchased from Bioresource Collection and Research Center (Hsinchu, Taiwan). The cells were cultured in Dulbecco’s modified Eagle’s medium (DMEM) containing 10% fetal bovine serum (FBS), 1% penicillin/streptomycin, and 1 mM sodium pyruvate at 37 °C in 5% CO_2_ incubator. Two days after confluence, the cells were cultured in FBS-containing DMEM with the incorporation of 500 μM 3-isobutyl-1-methylxanthine, 0.25 μM dexamethasone, and 10 μg/mL bovine insulin. After 2 days, the medium was changed to DMEM supplemented with 10% FBS and 10 μg/mL insulin. This was continued to day 8 when the preadipocytes had fully differentiated to adipocytes.

### 2.4. 3T3-L1 Cell Viability Assay

The preadipocytes were seeded in 96-well plates at a density of 1 × 10^5^ cells/well in DMEM. After 24 h, turmeric extract or curcuminoids in DMSO (0−40 μM) were added into the well for 24 h to examine the cytotoxicity. After this treatment, the cells were cultured with 3-(4,5-dimethylthiazol-2-yl)-2,5-diphenyltetrazolium bromide (MTT) at 5 μg/mL for a 4 h incubation. The unreacted dye was removed, and the formazan crystal was dissolved by dimethyl sulfoxide (100 μL). The absorbance was determined at 540 nm in an enzyme-linked immunosorbent assay (ELISA) reader. The differentiated 3T3-L1 cells were also used for detecting viability. The concentration of the differentiated cells for the viability assay was 2 × 10^4^ cells/well.

### 2.5. Oil Red O (ORO) Staining

Intracellular lipid accumulation in 3T3-L1 cells was detected using ORO staining. The preadipocytes were seeded in 6-well plates at a concentration of 8 × 10^4^ cells/well. The cells were treated with turmeric extract or curcuminoids dissolved in DMSO for 8 days. On day 8, the medium was removed, and the cells were washed using 20% isopropanol. Next, 10% formaldehyde was used to fix the cells for 1 h. The fixed cells were stained using ORO working solution for 20 min. After rinsing with double-distilled water, ORO was extracted using isopropanol (absorbance at 520 nm was measured spectrophotometrically).

### 2.6. Triglyceride (TG) Accumulation in Cells

The treatment and differentiation of 3T3-L1 cells were carried out using the same approach as for the ORO assay. Total amount of TG was quantified using a triglyceride quantification colorimetric/fluorometric kit (Biovision, Milpitas, CA, USA) based on the manufacturer’s protocol.

### 2.7. Protein Extraction and Western Blotting

The treatment and differentiation of 3T3-L1 cells were carried out using the same approach as for the ORO assay. The 3T3-L1 cells were collected and washed in PBS and lysed in a radioimmunoprecipitation assay buffer for 30 min on ice. The cell lysate was centrifuged at 12,000 rpm and 4 °C for 20 min. The supernatant was resolved in 12% sodium dodecyl sulfate polyacrylamide gel (SDS-PAGE), and then transferred to a nitrocellulose membrane. The blots were blocked in PBS containing 0.5% Tween 20 and 5% skim milk for 1 h, then incubated overnight at 4 °C with a polyclonal antibody against PPARγ (1:1000), C/EBPα (1:500), p-AMP-activated protein kinase α (p-AMPKα) (1:1000), ACC (1:1000), FAS (1:1000), or β-actin (1:10,000) (Abcam, Cambridge, UK). The membrane was incubated with horseradish peroxidase-conjugated secondary antibody (1:8000) and visualized using an enhanced chemiluminescence kit (PerkinElmer, Waltham, MA, USA).

### 2.8. ELISA Assay

The protein expression of interleukin (IL)-1β, IL-6, tumor necrosis factor (TNF)-α, and leptin in the supernatant of the cell medium was measured using ELISA kits (BioLegend, San Diego, CA, USA) according to manufacturer’s instructions. The absorbance was determined at 450 nm using a microplate spectrophotometer. The concentration of these adipokines was estimated based on the corresponding curves.

### 2.9. Statistical Analysis

Statistical differences were measured using the one-way analysis of variance followed by Tukey’s multiple comparison test. The data distribution was checked by Kolmogorov–Smirnov test. The *p* values of 0.05, 0.01, and 0.001 were considered statistically significant.

## 3. Results

### 3.1. Determination of Turmeric Extract

**Figure 2:**
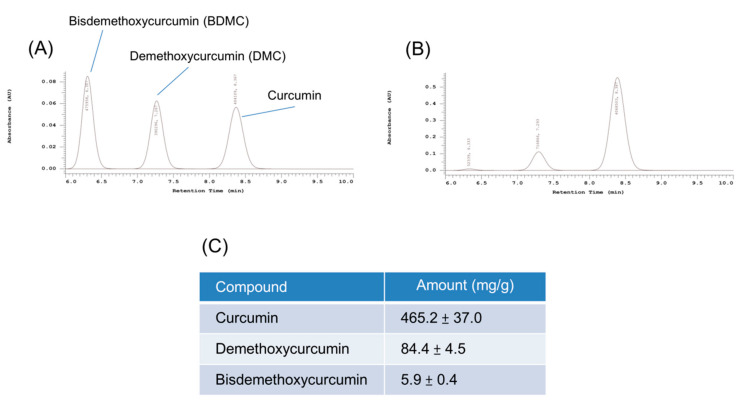
Quantitative analysis of curcumin, demethoxycurcumin (DMC), and bisdemethoxycurcumin (BDMC) in turmeric extract (BCM-95): (**A**) the HPLC chromatogram of the standards of three curcuminoids; (**B**) the HPLC chromatogram of three curcuminoids in turmeric extract; and (**C**) the amount (mg/g) of three curcuminoids in turmeric extract. All data are presented as the mean of three experiments ± S.E.M.

HPLC analysis was conducted for curcuminoid quantification in turmeric extract prior to the cell-based study. We firstly confirmed the retention time of standard curcumin, DMC, and BDMC, at 8.4, 7.3, and 6.3 min, respectively (Figure 2A). This indicated the lower lipophilicity of BDMC compared with other curcuminoids, as the shorter retention time suggests a less lipophilic compound. The representative HPLC profile of the turmeric extract indicated three peaks of curcuminoids (Figure 2B). The curcuminoid concentration in the extract was estimated with the established calibration curve (Figure 2C). The most abundant compound in the extract was curcumin (465.2 mg/g), followed by DMC (84.4 mg/g) and BDMC (5.9 mg/g).

### 3.2. Physicochemical Properties of Curcuminoids

The estimated physicochemical characteristics of curcumin, DMC, and BDMC were computed by molecular modeling, to understand the relationship with adipogenesis inhibition. Curcumin is relatively unstable in neutral or alkaline solution, and generates degradation products, such as ferulic acid. A dimer of ferulic acid is curcumin, in which two ferulates are linked through a methylene moiety (Figure 1). Thus, the physicochemical properties and anti-adipogenic effect of ferulic acid was also examined. The molecular weight of the four compounds ranged between 194 (ferulic acid) and 368 (curcumin) Da (Table 1). The molecular volume, which was predicted by molecular modeling, reflects molecular size. A correlation was found between molecular volume and molecular weight. Besides retention time, the tendency of lipophilicity can be assessed by Alog *P*. The trend of Alog *P* was as follows: ferulic acid (1.67) < curcumin (3.55) < DMC (3.57) < BDMC (3.59); however, the Alog *P* of the three curcuminoids was approximate. The H-bond acceptor number was decreased following the increase in dimethoxy. Both BDMC and ferulic acid exhibited the H-bond acceptor number of four. All the compounds possessed two H-bond donor numbers.

### 3.3. Adipocyte Viability Treated by Curcuminoids

**Figure 3:**
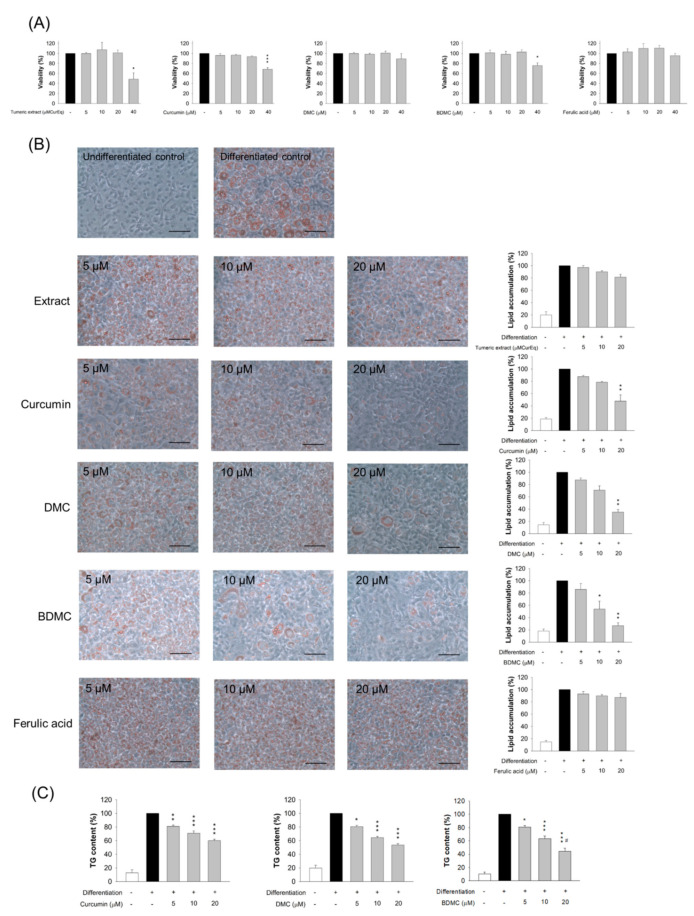
The 3T3-L1 cell viability and lipid accumulation after treatment of turmeric extract, curcuminoids, and ferulic acid. (**A**) The cell viability determined by MTT assay; (**B**) the ORO staining and the quantification of 3T3-LI cells; and (**C**) the intracellular TG content of 3T3-LI cells. All data are presented as the mean of three experiments ± S.E.M. *** *p* < 0.001; ** *p* < 0.01; * *p* < 0.05 as compared to the differentiated control group. ^#^ *p* < 0.05 as compared to the curcumin-treated group at the same dose.

MTT analysis was conducted in order to evaluate 3T3-L1 viability when treated with various concentrations of turmeric extract and the curcuminoids. The extract had negligible cytotoxicity within the range of 0−20 μM of the equivalent curcumin dose (Figure 3A). The adipocytes that were treated with 40 μM of the extract showed a significant decrease in viability compared with the controls. A similar result was observed for curcumin and BDMC. The cell viability following an intervention with 40 μM curcumin and BDMC was 68% and 76%, respectively. DMC and ferulic acid did not affect 3T3-L1 cell viability, even at the highest concentration of 40 μM. All the tested extracts or compounds, at doses of 0−20 μM, were considered non-cytotoxic to the cells. Concentrations of 5, 10, and 20 μM were employed for subsequent studies.

### 3.4. The Effect of Turmeric Extract and Curcuminoids on Lipid Accumulation

We next examined the effect of turmeric extract and curcuminoids on adipocyte differentiation, as determined by ORO staining and TG content. The preadipocytes differentiated into adipocytes for eight days, in the presence or absence of extract or curcuminoids. The intracellular lipids were stained by ORO. Based on the microscopic images (Figure 3B), the cells appeared to be round, with accumulated cytoplasmic lipid droplets. Lipid accumulation was estimated spectrophotometrically. Although the lipid accumulation was reduced following the extract treatment at higher concentrations, this inhibition did not achieve statistical significance (the right panel of Figure 3B). The number of intracellular lipid droplets was reduced when curcumin, DMC, or BDMC was present. The pure compounds arrested lipid deposition more profoundly than the corresponding concentration of the extract. Further, 5 μM of curcuminoid had no significant inhibitory effect on lipid accumulation. At 10 μM, a significant effect was observed with BDMC, but not curcumin and DMC. Concentrations up to 20 μM of all the curcuminoids resulted in a significant suppression of lipid accumulation compared with the controls. Lipid accumulation was decreased by 48%, 35%, and 27% with 20 μM curcumin, DMC, and BDMC, respectively. Ferulic acid did not inhibit adipocyte differentiation.

We also exposed 3T3-L1 cells to the extract or compounds after the induction of differentiation. All the tested materials demonstrated no cytotoxicity against mature adipocytes at concentrations of 0−20 μM (Figure S1A). It was found that 40 μM of extract caused a cytotoxicity of nearly 100%. The compounds exhibited minor cytotoxicity compared with the extract in the mature 3T3-L1 cells. Lipid accumulation was unaffected when the mature adipocytes were treated with the extract and compounds (Figure S1B). This result demonstrates that the curcuminoids blocked lipid accumulation only when applied at the onset of differentiation. We then assessed whether a pretreatment of curcumin, DMC, or BDMC on preadipocytes reduced TG. The turmeric extract and ferulic acid were excluded in this experiment, because of the observed minimal effect on lipid accumulation inhibition. The TG amount was decreased following the increase in curcuminoid concentration (Figure 3C). Of the three compounds tested, BDMC displayed the greatest inhibition of TG. TG in 3T3-L1 cells was reduced by 40%, 46%, and 56% when curcumin, DMC, and BDMC were added to the differentiation medium at a concentration of 20 μM, respectively.

### 3.5. The Effect of Curcuminoids on Expression of Adipogenic and Lipogenic Enzymes

**Figure 4:**
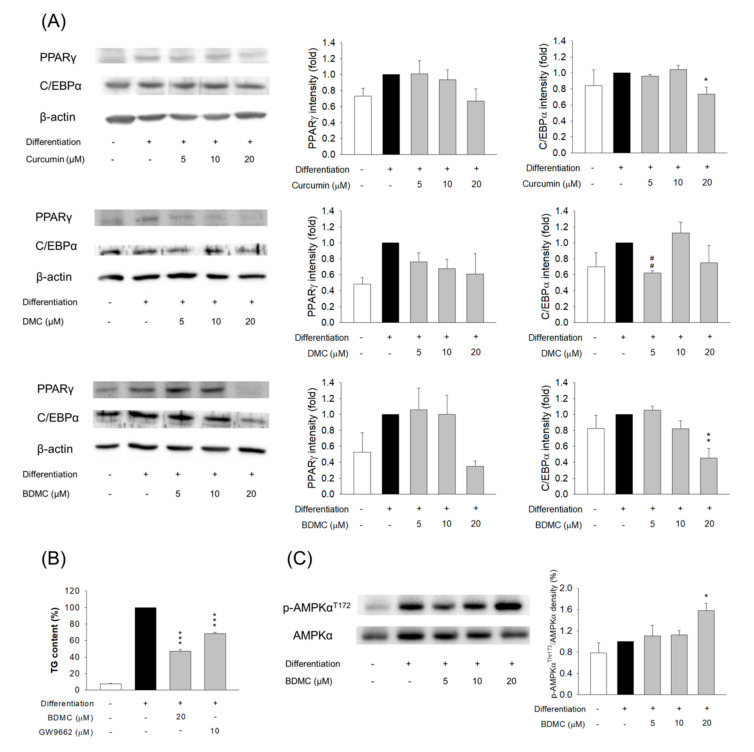
The effect of curcuminoids on transcription factors of 3T3-L1 cells. (**A**) The protein expression of PPARγ and C/EBPα after treatment of curcuminoids at 0−20 μM; (**B**) the intracellular TG content of 3T3-L1 cells after the treatment of BDMC and/or GW9662; and (**C**) the protein expression and AMPKα after treatment of BDMC at 0−20 μM. All data are presented as the mean of three experiments ± S.E.M. *** *p* < 0.001; ** *p* < 0.01; * *p* < 0.05 as compared to the differentiated control group. ^##^ *p* < 0.01 as compared to the curcumin-treated group at the same dose.

We sought to investigate the pathways through which curcuminoids inhibited adipogenesis. To explore the impact of curcuminoids on 3T3-L1 differentiation, by downregulating the adipogenic transcription factors PPARγ and C/EBPα, the protein expression was determined. PPARγ is a nuclear hormone receptor that is expressed during adipocyte differentiation, prior to C/EBPα. PPARγ and C/EBPα were elevated during adipocyte differentiation in the absence of curcuminoids. PPARγ was decreased in differentiated 3T3-L1 cells that had been treated with curcuminoids at 20 μM; however, statistical significance was only reached in the BDMC-treated group (Figure 4A). Curcumin at 20 μM considerably expressed less C/EBPα compared with the differentiated controls. DMC at 5 μM showed higher potency to inhibit C/EBPα compared with curcumin. Nevertheless, this effect was not observed at concentrations of 10 and 20 μM. BDMC at 5 and 10 μM appeared to have no effect on C/EBPα inhibition. BDMC at 20 μM significantly decreased the expression of C/EBPα by 55%. These results indicate that curcuminoids mediated adipocyte differentiation by suppressing adipogenic enzymes.

To verify the hypothesis that curcuminoids exerted anti-adipogenic activity through the modulation of transcription factors, a PPARγ inhibitor, GW9662, was used to treat the cells, in order to examine the TG levels. PPARγ inhibition by GW9662 markedly attenuated the TG content in the cells (Figure 4B). This result suggests the involvement of PPARγ regulation in the production of TG. We also found a significant decrease in TG, by nearly 50%, following the treatment of BDMC. The intervention of a PPARγ inhibitor in the BDMC-treated cells did not impact the TG levels, suggesting that factors other than transcription factors controlled adipogenesis inhibition by BDMC. AMPKα is a regulator of energy metabolism. The phosphorylation of AMPKα causes anti-adipogenesis by ACC suppression. We probed whether BDMC modulated AMPK phosphorylation. An increase in phospho-AMPKα after treatment of BDMC at 20 μM was observed (Figure 4C), indicating that BDMC modulated lipid metabolism, in part due to AMPKα activation.

**Figure 5:**
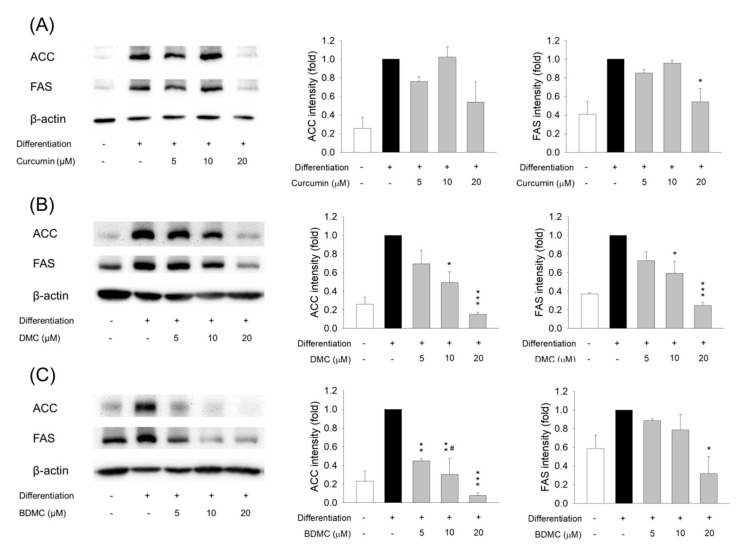
The effect of curcuminoids on lipogenic enzymes of 3T3-L1 cells. (**A**) The protein expression of ACC and FAS after treatment of curcumin at 0−20 μM; (**B**) the protein expression of ACC and FAS after treatment of DMC at 0−20 μM; and (**C**) the protein expression of ACC and FAS after treatment of BDMC at 0−20 μM. All data are presented as the mean of three experiments ± S.E.M. *** *p* < 0.001; ** *p* < 0.01; * *p* < 0.05 as compared to the differentiated control group. ^#^ *p* < 0.05 as compared to the curcumin-treated group at the same dose.

We next explored the protein level of lipogenic genes in the curcuminoid-treated 3T3-L1 cells. ACC is a rate-limiting enzyme in the synthesis of fatty acids. We found a notable increase in ACC after adipocyte differentiation (Figure 5A). Curcumin at 5 and 10 μM did not significantly change the overexpression of ACC, whereas a 20 μM dose lowered the ACC expression. A similar trend was observed for FAS downregulation by curcumin. DMC caused the inhibition of ACC and FAS in a dose-dependent manner (Figure 5B). BDMC showed greater inhibition on ACC than curcumin and DMC (Figure 5C). BDMC reduced ACC upregulation in differentiated cells by 92% at a 20 μM dose. This result suggested the involvement of lipogenic pathways in reduced differentiation by curcuminoids.

### 3.6. The Effect of Curcuminoids on Expression of Adipokines

**Figure 6:**
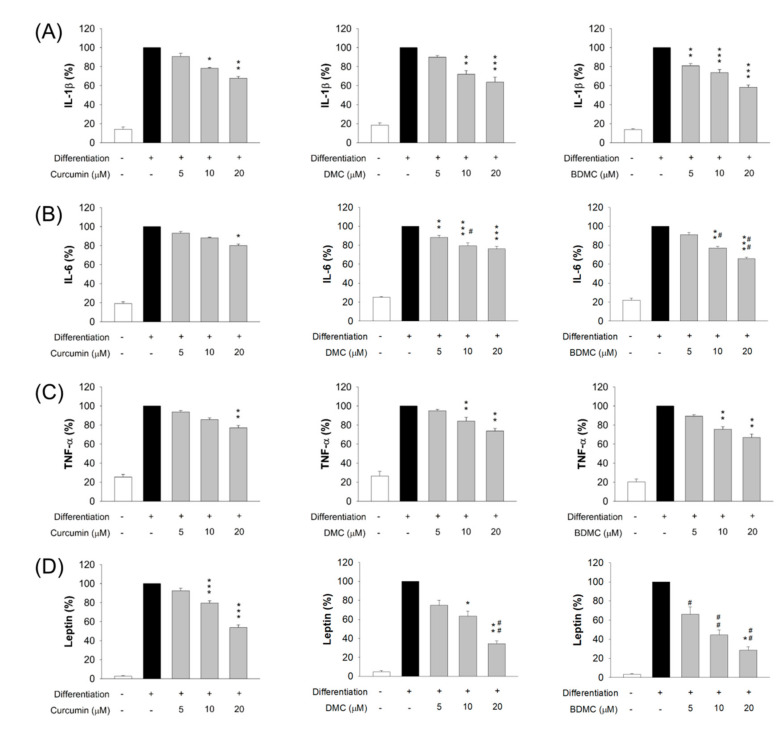
The effect of curcuminoids on adipokines of 3T3-L1 cells. (**A**) The protein expression of IL-1β after treatment of curcuminoids at 0−20 μM; (**B**) the protein expression of IL-6 after treatment of DMC at 0−20 μM; (**C**) the protein expression of IL-1β after treatment of BDMC at 0−20 μM; and (**D**) the protein expression of leptin after treatment of curcuminoids at 0−20 μM. All data are presented as the mean of three experiments ± S.E.M. *** *p* < 0.001; ** *p* < 0.01; * *p* < 0.05 as compared to the differentiated control group. ^##^ *p* < 0.01; ^#^ *p* < 0.05 as compared to the curcumin-treated group at the same dose.

We evaluated whether adipokine expression by adipocytes was influenced by curcuminoids. These adipokines included IL-1β, IL-6, TNF-α, and leptin. Markedly increased protein levels of the adipokines were found in differentiated adipocytes (Figure 6), with 7-, 5-, 4-, and 40-fold increases in IL-1β, IL-6, TNF-α, and leptin, after differentiation, as compared to the non-differentiation control group, respectively. These increases were reduced by curcuminoids, at concentrations of 5−20 μM. A similar reduction in the protein level of the proinflammatory cytokine IL-1β was observed after the treatment of three compounds (Figure 6A). IL-6 inhibition was increased in the order of curcumin < DMC < BDMC (Figure 6B). The expression of IL-6 in the cells was decreased to 80%, 76%, and 66% by curcumin, DMC, and BDMC at a concentration of 20 μM, respectively. A similar trend was shown following the inhibition of TNF-α, although the difference in suppression by these curcuminoids did not attain statistical significance (Figure 6C). Leptin protein expression was arrested, in a concentration-dependent manner, by the curcuminoids, notably BDMC (Figure 6D). BDMC at 20 μM reduced leptin by 72% compared with differentiated cells without the treatment.

### 3.7. Molecular Docking of Curcuminoids on PPARγ, C/EBPα, and ACC

**Figure 7:**
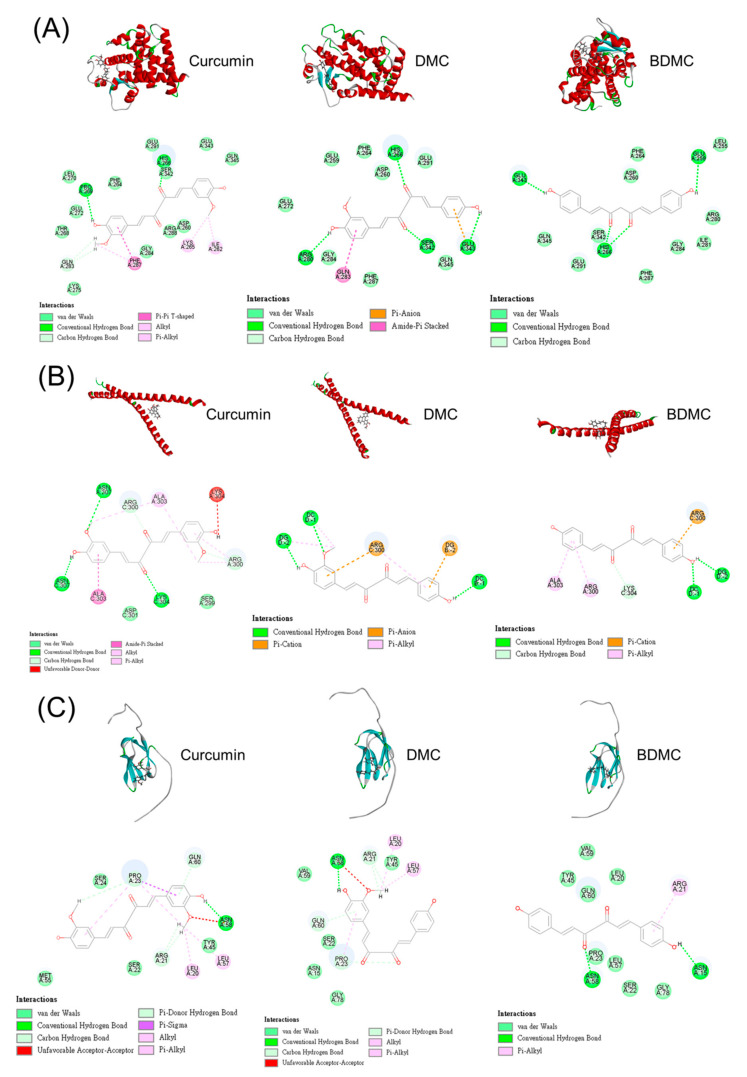
The docking poses of curcuminoids at the adipogenic or lipogenic factors. (**A**) The docking poses and the interaction of curcuminoids at PPARγ; (**B**) the docking poses and the interaction of curcuminoids at C/EBPα; and (**C**) the docking poses and the interaction of curcuminoids at ACC.

We conducted molecular docking to elucidate the possible mechanism between curcuminoids and adipogenic or lipogenic markers, such as PPARγ, C/EBPα, and ACC. The three-dimensional model of these proteins was generated for their posterior use as the target structure in the in silico computation (the upper panel of Figure 7A–C). The possible interaction between compounds and proteins is illustrated in the lower panel of Figure 7A–C. The hydrogen bonding and van der Waals force were established by PPARγ and interacted with curcuminoids (Figure 7A). The docking result of PPARγ and curcumin showed that the Pi–Pi T-shaped interaction was stacked to Lys265. The Pi–anion and amide–Pi interactions were formed between the aromatic rings of DMC and PPARγ. However, these interactions were absent in BDMC. The lowest binding energy score (negative CDOCKER energy) of the curcuminoids interacting with proteins was calculated (Table 2). The greater negative energy indicates a stronger binding interaction. We demonstrated that PPARγ interacted with DMC with the strongest force (-22), followed by BDMC (-18), and curcumin (-17).

With respect to C/EBPα, the cooperativity of the interaction with curcuminoids included nan der Waals, hydrogen bonding, Pi–anion, Pi–cation, and Pi–alkyl forces (Figure 7B). BDMC (-34) displayed the highest negative CDOCKER score with the interaction with C/EBPα, followed by DMC (-31), and curcumin (-28). This result demonstrated that BDMC could dock into a C/EBPα dimer in order to establish a stable complex. Although the interaction types between ACC and BDMC were fewer than those of curcumin and DMC (Figure 7C), BDMC demonstrated the greatest interaction with ACC (Table 2).

## 4. Discussion

Obesity is a primary risk factor for diabetes, cardiovascular disorders, and cancers, contributing to a reduced life expectancy of 5−20 years [3]. The prevalence of obesity has tripled since 1975, and continues to increase. Some natural compounds have been found to have a beneficial effect on obesity. Curcumin from turmeric is an anti-obesity compound that has drawn attention as a therapeutic agent, due to evidence that supports its role in adipogenesis inhibition. We currently compare the ability of curcumin and its demethoxy derivatives to inhibit 3T3-L1 differentiation and adipogenesis. Among the curcuminoids that have been isolated from turmeric extract, BDMC showed a stronger anti-adipogenic potency in 3T3-L1 cells compared with curcumin and DMC. The curcuminoids inhibited adipogenesis and lipogenesis via control of the expression of PPARγ, C/EBPα, ACC, FAS, and AMPKα. The adipokines released from the 3T3-L1 differentiated cells were also downregulated by these compounds. We predicted a possible interaction of curcuminoids with C/EBPα and ACC, according to the molecular docking profiles. The curcuminoids in turmeric extract contained 83.7% curcumin, 15.2% DMC, and 1.1% BDMC. This percentage was similar to the turmeric extracts that were produced by the other investigators (70%−80% curcumin, 15%−20% DMC, and 3%−5% BDMC) [11,12,14]. Nevertheless, the percentage of curcuminoids from the turmeric extract only accounted for one-half of the proportion in our study. The other components in the extract could be carbohydrates, proteins, fats, minerals, and essential oils [11]. In our study, the extract had a minimal effect on 3T3-L1 cell differentiation. This result may indicate that components other than curcuminoids offset the anti-adipogenic activity of curcuminoids in the extract.

Energy balance and lipid homeostasis are regulated by adipocytes. Adipocyte differentiation determines the number of adipocytes that have formed in the development of obesity. We employed 3T3-L1 cells as a model to investigate the effect of curcuminoids on the inhibition of adipocyte differentiation. The 3T3-L1 cells can spontaneously differentiate into adipocytes, by exposing them to a hormonal cocktail and high concentration of insulin. The suppression of adipogenesis can be due to both biochemical mechanisms behind anti-adipogenic activity and the simple lysis of adipocyte membranes [15]. We found that adipocyte viability was not significantly affected by curcuminoids at concentrations ≤ 20 μM, demonstrating that these compounds suppressed adipogenesis through means other than apoptosis induced by membrane lysis. Curcumin is reported to attenuate obesity-related disorders via different mechanisms, such as transcription factors, cellular receptors, growth factors, cytokines, and chemokines [16]. An increase in adipose tissue is generated by the increased size and number of lipid droplets in adipocytes [17]. Lipid droplets are dynamic cellular structures for transient stocks of lipids. An increase in lipid droplet size after adipocyte differentiation was observed. A large lipid droplet is associated with poor transport and metabolization of fatty acids in metabolically inert organelles [18]. In this study, three curcuminoids inhibited lipid accumulation in lipid droplets without cytotoxicity. BDMC was associated with the greatest potency to inhibit lipid accumulation, followed by DMC and curcumin. Although ferulic acid, the degradation product of curcumin, is reported to have antioxidant and anti-inflammatory activities [19], no suppression of lipid accumulation was detected. This indicates the importance of dimer structure for inhibiting adipocyte differentiation. The curcuminoids had no significant effect on lipid accumulation on the differentiated adipocytes, suggesting that the inhibition by curcuminoids primarily occurred in the differentiative stage. Curcuminoids may demonstrate preventive, but not therapeutic, efficacy for the management of obesity. TGs are the primary form of fatty acid storage in adipocytes and other organs. Enhanced intracellular TG deposition can contribute to the development of obesity [20]. We also found improved performance of BDMC on the inhibition of TG production compared with curcumin and DMC.

The C/EBP family is vital for adipocyte differentiation. The early induction of C/EBPδ in conjunction with C/EBPβ has demonstrated a crucial role in promoting the expression of PPARγ and C/EBPα, which are both key regulators of adipocyte differentiation [21]. C/EBPα functions to sustain PPARγ expression in a positive feedback mechanism. Curcumin was found to decrease TG, through decreased PPARγ and C/EBPα, in the early stages of differentiation [22,23]. We observed that BDMC inhibited the expression of PPARγ and C/EBPα proteins to a greater extent than curcumin. It has been suggested that both the transcription factors were required to inhibit differentiation by curcuminoids. ACC and FAS are PPARγ-targeted lipogenic factors [24]. Lipogenesis is the process of fatty acid synthesis, and the subsequent production and storage of TG [25]. FAS is a lipogenic enzyme that is associated with adipocyte differentiation, obesity development, and insulin resistance [26]. The present study demonstrated that DMC and BDMC attenuated adipocyte differentiation through regulating ACC and FAS proteins in a concentration-dependent manner.

The PPARγ inhibitor GW9662 could not completely reverse TG inhibition by BDMC, supporting the hypothesis that signaling pathways other than PPARγ and C/EBPα controlled BDMC suppression of adipogenesis. AMPK is an energy regulator and sensor in adipocytes. The activation of AMPK leads to the phosphorylation of ACC and FAS, resulting in the direct inhibition of malonyl-CoA and de novo lipogenesis [13]. ACC is a downstream effector of AMPK for lipid biosynthesis; thus, AMPK activation may also be involved in the repression of adipogenesis by curcuminoids. Given that BDMC promoted AMPK phosphorylation in 3T3-L1 cells, the anti-adipogenic activity was further mediated by the activation of AMPK. The involvement of AMPK signaling is important for BDMC, since AMPK activation is regarded as a target in the prevention of obesity [27]. The collective mechanisms of action are associated with the strong anti-adipogenic effect of BDMC.

In addition to lipid substances, adipocytes release a series of cytokines, which are collectively named adipokines. Lipid accumulation is associated with a low-grade inflammatory condition in adipose tissue. A link between obesity and inflammation has been established, and specifically the secretion of proinflammatory adipokines such as IL-1β, IL-6, TNF-α, and MCP-1 is increased [28]. Obesity-induced adipokines can influence the function of adipocytes, through the infiltration of immune cells, including macrophages and neutrophils, to form a vicious cycle [29]. We had detected the change in IL-1β, IL-6, and TNF-α in 3T3-L1 cells after the treatment with curcuminoids. Adipocytes initially express IL-1β to elicit the proinflammatory cytokines IL-6 and TNF-α [30]. The first indication of increased adipokine release in obesity is provided by the identification of increased TNF-α [31]. This cytokine contributes to the adipokine dysregulation in adipocytes, to correlate with the degree of adiposity, body mass index, body fat percentage, and insulin resistance [32]. Similarly to TNF-α, IL-6 levels increase with obesity and body fat [33]. The recruitment of macrophages in adipose tissue is associated with increased IL-6 and TNF-α secretion, leading to insulin resistance [34]. Curcumin is known as an anti-inflammatory molecule that inhibits cytokines [35]. The anti-inflammatory effect of curcumin has been demonstrated to be mediated by the downregulation of IL-1, IL-6, and TNF-α [36]. We showed that the demethoxy derivatives of curcumin were superior to the parent compound in the inhibition of adipokines, especially IL-6. Leptin is an adipose tissue hormone that regulates energy intake and balance. It can be categorized as a proinflammatory adipokine to stimulate the expression of IL-6 and TNF-α [37]. PPARγ and C/EBPα are the upstream actors that are necessary for the expression of leptin to regulate glucose and lipid metabolism [38]. Both DMC and BDMC had a greater impact on lessening leptin expression compared with curcumin. This result further supports the effect on adipogenesis inhibition, through PPARγ and C/EBPα modulation. Curcuminoids could counteract inflammation in adipocytes. It may be beneficial to attenuate the low-grade chronic inflammation that is associated with metabolic syndromes.

Our data showed greater anti-adipogenic activity of demethoxy derivatives compared with curcumin. This illustrated that the methoxy groups on the phenyl ring play an essential role in the control of adipocyte differentiation. The anti-adipogenic activity of curcuminoids was quite different than that of other bioactivities. The antioxidant activity of curcuminoids, determined by the linoleic acid peroxidation approach, represents a trend of BDMC < DMC < curcumin [39]. A similar tendency has been observed in the inhibitory capacity on supercoiled plasma DNA damage [40]. A previous study [41] has reported that curcuminoids suppress TNF-α-induced NF-κB activation in the order of BDMC < DMC < curcumin. The anti-breast cancer and anti-melanoma activities of BDMC were also less than curcumin and DMC in a cell-based study [42]. Although these investigations exhibited items to the contrary of our study, the superior chemical stability of BDMC over curcumin and DMC could be the reason for the observed stronger anti-adipogenic effect. Another possibility could be that the methoxy moieties in the curcumin structure might contribute a steric hindrance for cell membrane penetration and receptor binding. Matsuda et al. [43] reported that the flavonoids with methoxy groups are beneficial to enhance TG accumulation in 3T3-L1 cells. We may infer that curcumin and DMC containing methoxy groups were unfavorable to suppress adipogenesis, based on the case of flavonoids. The smaller molecular size of BDMC may also be favorable to penetrate across the adipocyte membrane, leading to cell uptake. Further work is needed to elucidate the correlation between the chemical features of curcuminoids and anti-adipogenic activity.

Curcumin can directly interact with a wide range of biomolecules, such as transcription factors, cellular receptors, and enzymes, for attenuating obesity-related disorders via different signaling mechanisms [16,44]. Curcumin is proved to inhibit the mRNA level of adipogenic transcription factors, particularly PPARγ and C/EBPα, in the early stage of adipocyte differentiation [45]. We employed molecular docking to predict possible interactions between curcuminoids and adipogenic factors, including PPARγ, C/EBPα, and ACC. Although the structures of the three curcuminoids exhibited similar backbones, the interaction with these proteins was quite different. The demethoxy derivatives showed stronger interactions with all the proteins that were tested, compared with curcumin. This confirmed the results from the cell-based study that showed DMC and BDMC usually possessed higher anti-adipogenic activity as compared to curcumin. A strongest interaction with C/EBPα and ACC was found between BDMC, followed by DMC and curcumin. A similar tendency was detectable in the Western blot assay of C/EBPα and ACC. The involvement of the C/EBPα and ACC pathways in curcuminoid-treated adipocytes was validated. This also suggested that curcuminoids might directly interact with C/EBPα and ACC, to inhibit adipocyte differentiation.

**Figure 8:**
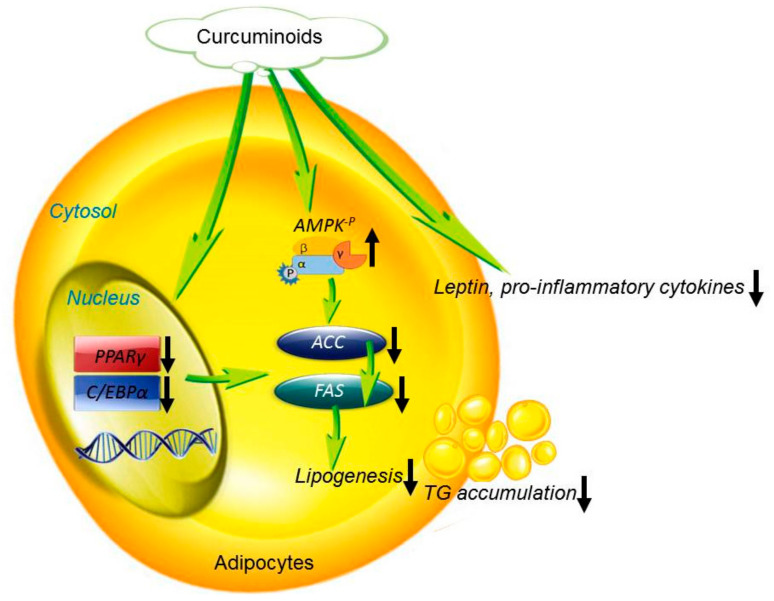
The proposed mechanisms of the anti-adipogenic activity of curcuminoids.

We currently demonstrated that BDMC inhibited 3T3-L1 differentiation, mainly through the downregulation of PPARγ and C/EBPα, leading to a subsequent downregulation of their target proteins ACC and FAS (Figure 8). Thus, lipid accumulation was inhibited by this pathway. BDMC also enhanced AMPK phosphorylation and reduced the expression of the downstream molecules ACC and FAS. Lipid accumulation was decreased by these pathways, resulting in a counteracting of proinflammatory adipokines. Given that humans can consume up to 8 g of curcumin per day, without experiencing adverse effects [46], curcuminoids are an interesting preventive intervention against obesity and other related diseases. Due to the anti-adipogenic activity of BDMC in this study, we expect that BDMC will be a potential therapy for obesity management. Though curcumin is considered safe after administration in humans, some clinical studies have shown limited therapeutic effectiveness, due to its instability and poor oral bioavailability [47,48]. Since both DMC and BDMC are more chemically stable than curcumin in a physiological medium [49], the demethoxy derivatives may have applicability for preventing obesity.

## 5. Conclusions

We demonstrated that curcuminoids inhibited 3T3-L1 cell differentiation, and that BDMC was the most active compound responsible for this effect. These compounds displayed anti-adipogenic activity, by reducing the expression of PPARγ and C/EBPα, which are the main transcription factors of adipogenesis in adipocyte differentiation. In addition, AMPKα phosphorylation was activated by curcuminoids, leading to the reduction in ACC and FAS expression. The subsequent TG generation and adipokine release were decreased. These results suggested that curcuminoids inhibit lipid accumulation via modulation of adipogenic transcription factors and lipogenic enzymes during differentiation for hindering preadipocyte maturation. Although BDMC revealed the greatest potential to inhibit adipogenesis among the three compounds tested, the inhibitory effect of the turmeric extract was insignificant, due to the very low amount of BDMC in the extract. It is important to select the turmeric products with abundant BDMC content for presenting considerable anti-adipogenic efficiency. The anti-adipogenic application of curcuminoids could be preventive. Our data suggest the potential of BDMC for improving obesity and the related chronic inflammation. Further studies are needed to further examine these expectations.

## Figures and Tables

**Table 1:** Physicochemical properties of curcumin and its derivatives determined by molecular modeling.

Physicochemical Property	Curcumin	DMC	BDMC	Ferulic Acid
Molecular formula	C_21_H_20_O_6_	C_20_H_18_O_5_	C_19_H_16_O_4_	C_10_H_10_O_4_
Molecular weight (Da)	368.38	338.35	308.33	194.18
Molecular volume (Å^3^)	295.66	267.88	241.27	151.60
Alog *P*	3.55	3.57	3.59	1.67
Hydrogen bond acceptor number	6	5	4	4
Hydrogen bond donor number	2	2	2	2

Alog *P*, predicted oil/water partition coefficient; BDMC, bisdemethoxycurcumin; DMC, demethoxycurcumin.

**Table 2:** The negative CDOCKER energy of curcumin and its derivatives with PPARγ, C/EBPα, and ACC.

Protein (PDB Code)	Curcumin	DMC	BDMC
PPARγ (1ZGY)	−16.757	−22.056	−18.371
C/EBPα (1NWQ)	−27.946	−31.258	−34.034
ACC (2DN8)	−12.264	−20.809	−22.749

BDMC, bisdemethoxycurcumin; DMC, demethoxycurcumin.

## Supplementary Materials

The following are available online at https://www.mdpi.com/article/10.3390/biom11071025/s1, Figure S1: The viability and lipid accumulation of the 3T3-L1 cell exposed to the extract or compounds after the induction of differentiation. All the tested materials demonstrated no cytotoxicity against mature adipocytes at concentrations of 0−20 μM (Figure S1A). Lipid accumulation was unaffected when the mature adipocytes were treated with the extract and compounds (Figure S1B).

## References

[1] Chooi Y.C., Ding C., Magkos F. (2019). The epidemiology of obesity. Metab. Clin. Exp..

[2] Bray G.A., Frühbeck G., Ryan D.H., Wilding J.P.H. (2016). Management of obesity. Lancet.

[3] Blüher M. (2019). Obesity: Global epidemiology and pathogenesis. Nat. Rev. Endocrinol..

[4] Longo M., Zatterale F., Naderi J., Parrillo L., Formisano P., Raciti G.A., Beguinot F., Miele C. (2019). Adipose tissue dysfunction as determinant of obesity-associated metabolic complications. Int. J. Mol. Sci..

[5] Kang J.G., Park C.Y. (2012). Anti-obesity drugs: A review about their effects and safety. Diabetes Metab. J..

[6] Karri S., Sharma S., Hatware K., Patil K. (2019). Natural anti-obesity agents and their therapeutic role in management of obesity: A future trend perspective. Biomed. Pharmacol..

[7] Rolfe V., Mackonochie M., Mills S., MacLennan E. (2020). Turmeric/curcumin and health outcomes: A meta-review of systematic reviews. Eur. J. Integr. Med..

[8] Tsuda T. (2018). Curcumin as a functional food-derived factor: Degradation products, metabolites, bioactivity, and future perspectives. Food Funct..

[9] Bradford P.G. (2013). Curcumin and obesity. BioFactors.

[10] Mohammadi A., Sahebkar A., Iranshahi M., Amini M., Khojasteh R., Ghayour-Mobarhan M., Ferns G.A. (2013). Effects of supplementation with curcuminoids on dyslipidemia in obese patients: A randomized crossover trial. Phytother. Res..

[11] Panahi Y., Khalili N., Hosseini M.S., Abbasinazari M., Sahebkar A. (2014). Lipid-modifying effects of adjunctive therapy with curcuminoids-piperine combination in patients with metabolic syndrome: Results of a randomized controlled trial. Complent. Ther. Med..

[12] Lai C.S., Chen Y.Y., Lee P.S., Kalyanam N., Ho C.T., Liou W.S., Yu R.C., Pan M.H. (2016). Bisdemethoxycurcumin inhibits adipogenesis in 3T3-L1 preadipocytes and suppresses obesity in high-fat diet-fed C57BL/6 mice. J. Agric. Food Chem..

[13] Song Z., Xiaoli A.M., Yang F. (2018). Regulation and metabolic significance of *de novo* lipogenesis in adipose tissues. Nutrients.

[14] Gordon O.N., Luis P.B., Ashley R.E., Osheroff N., Schneider C. (2015). Oxidative transformation of demethoxy- and bisdemethoxycurcumin: Products, mechanism of formation, and poisoning of human topoisomerase IIα. Chem. Res. Toxicol..

[15] Jakab J., Miškić B., Mikšić Š., Juranić B., Ćosić V., Schwarz D., Včev A. (2021). Adipogenesis as a potential anti-obesity target: A review of pharmacological treatment and natural products. Diabetes Metab. Syndr. Obes..

[16] Varì R., Scazzocchio B., Silenzi A., Giovannini C., Masella R. (2021). Obesity-associated inflammation: Does curcumin exert a beneficial role?. Nutrients.

[17] Onal G., Kutlu O., Gozuacik D., Emre S.D. (2017). Lipid droplets in health and disease. Lipids Health Dis..

[18] Somwar R., Roberts C.T., Varlamov O. (2011). Live-cell imaging demonstrates rapid cargo exchange between lipid droplets in adipocytes. FEBS. Lett..

[19] De Oliveira Silva E., Batista R. (2017). Ferulic acid and naturally occurring compounds bearing a feruloyl moiety: A review on their structures, occurrence, and potential health benefits. Compr. Rev. Food Sci. Food Saf..

[20] Alves-Bezerra M., Cohen D.E. (2018). Triglyceride metabolism in the liver. Compr. Physiol..

[21] Sarantopoulos C., Banyard D.A., Ziegler M.E., Sun B., Shaterian A., Widgerow A.D. (2018). Elucidating the preadipocyte and its role in adipocyte formation: A comprehensive review. Stem Cell Rev. Rep..

[22] Pan Y., Zhao D., Yu N., An T., Miao J., Mo F., Gu Y., Zhang D., Gao S., Jiang G. (2017). Curcumin improves glycolipid metabolism through regulating peroxisome proliferator activated receptor γ signaling pathway in high-fat diet-induced obese mice and 3T3-L1 adipocytes. R. Soc. Open Sci..

[23] Sakuma S., Sumida M., Endoh Y., Kurita A., Yamaguchi A., Watanabe T., Kohda T., Tsukiyama Y., Fujimoto Y. (2017). Curcumin inhibits adipogenesis induced by benzyl butyl phthalate in 3T3-L1 cells. Toxicol. Appl. Pharmacol..

[24] Tung Y.C., Hsieh P.H., Pan M.H., Ho C.T. (2017). Cellular models for the evaluation of the antiobesity effect of selected phytochemicals from food and herbs. J. Food Drug Anal..

[25] Wallace M., Metallo C.M. (2020). Tracing insights into *de novo* lipogenesis in liver and adipose tissues. Semin. Cell Dev. Biol..

[26] Moseti D., Regassa A., Kim W.K. (2016). Molecular regulation of adipogenesis and potential anti-adipogenic bioactive molecules. Int. J. Mol. Sci..

[27] Fang C., Kim H., Noratto G., Sun Y., Talcott S.T., Mertens-Talcott S.U. (2018). Gallotanin derivatives from mango (*Mangifera indica* L.) suppress adipogenesis and increase thermogenesis in 3T3-L1 adipocytes in part through the AMPK pathway. J. Funct. Foods.

[28] Taylor E.B. (2021). The complex role of adipokines in obesity, inflammation, and autoimmunity. Clin. Sci..

[29] Francisco V., Pino J., Gonzalez-Gay M.A., Mera A., Lago F., Gómez R., Mobasheri A., Gualillo O. (2018). Adipokines and inflammation: Is it a question of weight?. Br. J. Pharmacol..

[30] Zieger K., Weiner J., Krause K., Schwarz M., Kohn M., Stumvoll M., Blüher M., Heiker J.T. (2018). Vaspin suppresses cytokine-induced inflammation in 3T3-L1 adipocytes via inhibition of NFκB pathway. Mol. Cell. Endocrinol..

[31] Sung J.H., Chon J.W., Lee M.A., Park J.K., Woo J.T., Park Y.K. (2011). The anti-obesity effect of *Lethariella dadonioides* in 3T3-L1 cells and obese mice. Nutr. Res. Pract..

[32] Bai Y., Sun Q. (2015). Macrophage recruitment in obese adipose tissue. Obes. Rev..

[33] Wensveen F.M., Valentić S., Šestan M., Wensveen T.T., Polić B. (2015). The “big bang” in obese fat: Events initiating obesity-induced adipose tissue inflammation. Eur. J. Immunol..

[34] Stolarczyk E. (2017). Adipose tissue inflammation in obesity: A metabolic or immune response?. Curr. Opin. Pharmacol..

[35] Marton L.T., Barbalho S.M., Sloan K.P., Sloan L.A., de Alvares Goulart R., Araújo A.C., Bechara M.D. (2020). Curcumin, autoimmune and inflammatory diseases: Going beyond conventional therapy−a systematic review. Crit. Rev. Food Sci. Nutr..

[36] Simental-Mendía L.E., Cicero A.F.G., Atkin S.L., Majeed M., Sahebkar A. (2019). A systematic review and meta-analysis of the effect of curcuminoids on adiponectin levels. Obes. Res. Clin. Pract..

[37] Jayarathne S., Stull A.J., Miranda A., Scoggin S., Claycombe-Larson K., Kim J.H., Moustaid-Moussa N. (2018). Tart cherry reduces inflammation in adipose tissue of zucker fatty rats and cultured 3T3-L1 adipocytes. Nutrients.

[38] Lowe C.E., O’Rahilly S., Rochford J.J. (2011). Adipogenesis at a glance. J. Cell Sci..

[39] Jayaprakasha G.K., Rao L.J., Sakariah K.K. (2006). Antioxidant activities of curcumin, demethoxycurcumin and bisdemethoxycurcumin. Food Chem..

[40] Ahsan H., Parveen N., Khan N.U., Hadi S.M. (1999). Pro-oxidant, anti-oxidant and cleavage activities on DNA of curcumin and its derivatives demethoxycurcumin and bisdemethoxycurcumin. Chem.-Biol. Interact..

[41] Sandur S.K., Pandey M.K., Sung B., Ahn K.S., Murakami A., Sethi G., Limtrakul P., Badmaev V., Aggarwal B.B. (2007). Curcumin, demethoxycurcumin, bisdemethoxycurcumin, tetrahydrocurcumin and turmerones differentially regulate anti-inflammatory and anti-proliferative responses through a ROS-independent mechanism. Carcinogenesis.

[42] Huang C., Lu H.F., Chen Y.H., Chen J.C., Chou W.H., Huang H.C. (2020). Curcumin, demethoxycurcumin, and bisdemethoxycurcumin induced caspase-dependent and –independent apoptosis via Smad or Akt signaling pathways in HOS cells. BMC Complement. Med. Ther..

[43] Matsuda H., Kogami Y., Nakamura S., Sugiyama T., Ueno T., Yoshikawa M. (2011). Structural requirements of flavonoids for the adipogenesis of 3T3-L1 cells. Bioorg. Med. Chem..

[44] Shehzad A., Ha T., Subhan F., Lee Y.S. (2011). New mechanisms and anti-inflammatory role of curcumin in obesity and obesity-related metabolic diseases. Eur. J. Nutr..

[45] Kim C.Y., Young C., Le T.T., Chen C., Cheng J.X., Kim K.H. (2011). Curcumin inhibits adipocyte differentiation through modulation of mitotic clonal expansion. J. Nutr. Biochem..

[46] Cheng A.L., Hsu C.H., Lin J.K., Hsu M.M., Ho Y.F., Shen T.S., Ko J.Y., Lin J.T., Lin B.R., Wu M.S. (2001). Phase I clinical trial of curcumin, a chemopreventive agent, in patients with high-risk or pre-malignant lesions. Anticancer Res..

[47] Wickenberg J., Ingemansson S.L., Hlebowicz J. (2010). Effects of *Curcuma longa* (turmeric) on postprandial plasma glucose and insulin in healthy subjects. Nutr. J..

[48] Yang Y.S., Su Y.F., Yang H.W., Lee Y.H., Chou J.I., Ueng K.C. (2014). Lipid-lowering effects of curcumin in patients with metabolic syndrome: A randomized, double-blind, placebo-controlled trial. Phytother. Res..

[49] Bettini S., Vergara D., Bonsegna S., Giotta L., Toto C., Chieppa M., Maffia M., Giovinazzo G., Valli L., Santino A. (2013). Efficient stabilization of natural curcuminoids mediated by oil body encapsulation. RSC Adv..

