# Supplementary figures and images for: The Demethoxy Derivatives of Curcumin Exhibit Greater Differentiation Suppression in 3T3-L1 Adipocytes Than Curcumin: A Mechanistic Study of Adipogenesis and Molecular Docking
Source: Biomolecules. 2021 Jul 14;11(7):1025. doi: 10.3390/biom11071025 (PMC8301910; doi:10.3390/biom11071025)

(A)

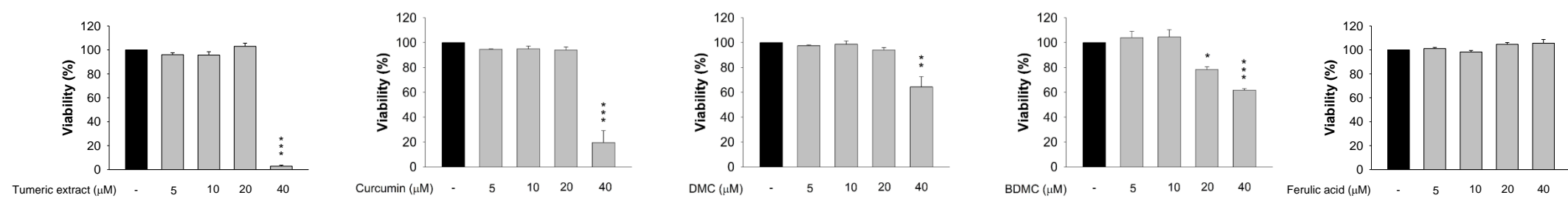

(B)

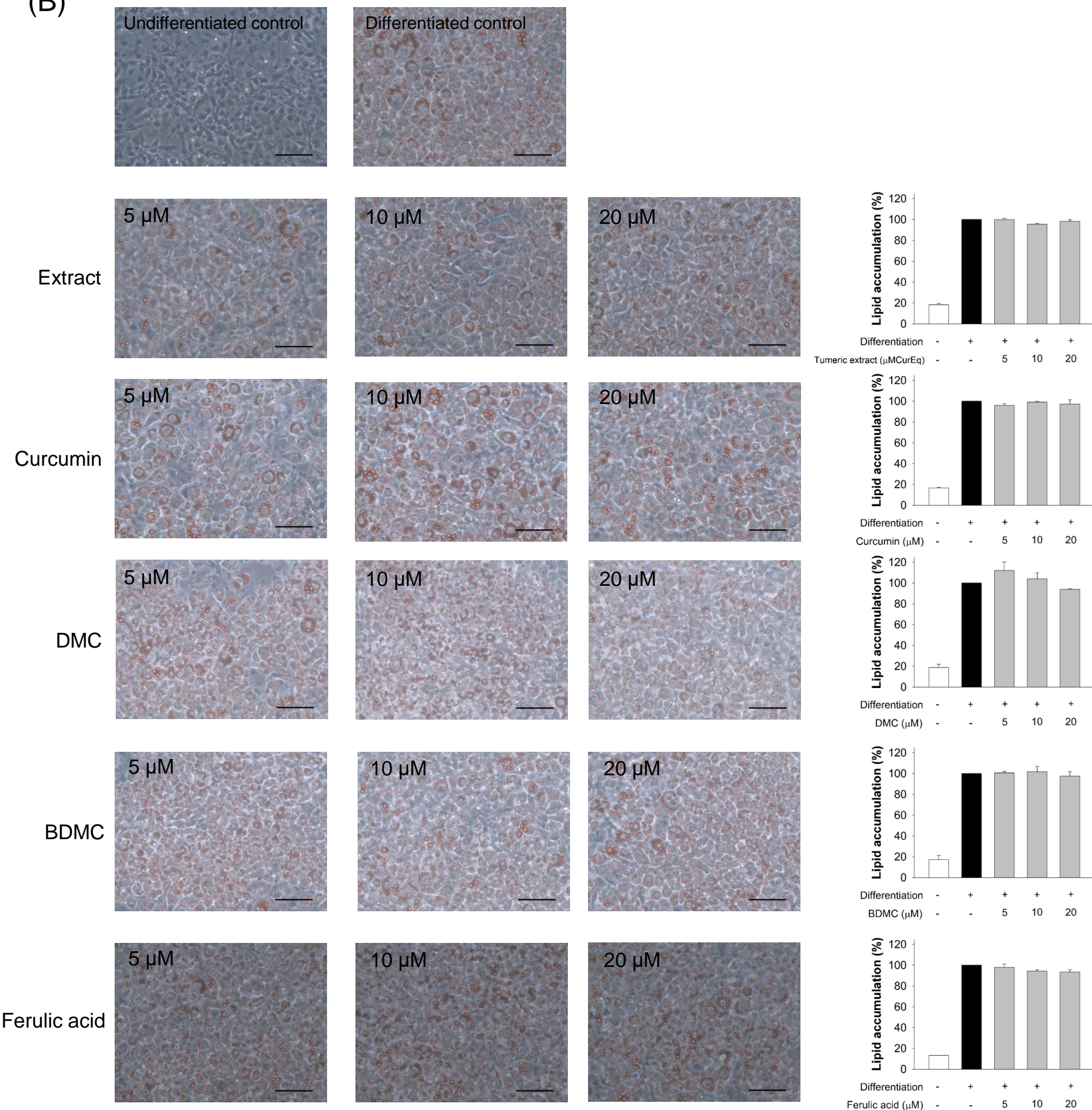

Suppl. Fig. 1

Supplement: Supplementary file 1 [file biomolecules-11-01025-s001.zip › biomolecules-1287537-supplementary.pdf]
